# Parameter Study of Superabsorbent Polymers (SAPs) for Use in Durable Concrete Structures

**DOI:** 10.3390/ma12091541

**Published:** 2019-05-10

**Authors:** Laurence De Meyst, Els Mannekens, Maria Araújo, Didier Snoeck, Kim Van Tittelboom, Sandra Van Vlierberghe, Nele De Belie

**Affiliations:** 1 Magnel Laboratory for Concrete Research, Department of Structural Engineering, Ghent University, Tech Lane Ghent Science Park, Campus A, Technologiepark Zwijnaarde 60, B-9052 Ghent, Belgium; Laurence.DeMeyst@UGent.be (L.D.M.); Adelaide.Araujo@UGent.be (M.A.); Didier.Snoeck@UGent.be (D.S.); Kim.VanTittelboom@UGent.be (K.V.T.); 2 ChemStream bvba, Drie Eikenstraat 661, B-2650 Edegem, Belgium; Els.Mannekens@Chemstream.be; 3 Polymer Chemistry and Biomaterials Group, Centre of Macromolecular Chemistry, Department of Organic and Macromolecular Chemistry, Ghent University, Krijgslaan 281, Building S4-bis, 9000 Ghent, Belgium; Sandra.VanVlierberghe@UGent.be

**Keywords:** superabsorbent polymers (SAPs), mortar properties, particle size, cross-linker, solubles

## Abstract

Superabsorbent polymers (SAPs) can be added to a concrete mixture to provide internal curing and reduce the risk for early-age shrinkage cracking. Hence, they can help to increase the overall durability of concrete structures. The type, swelling characteristics, kinetics of water release, amount and particle size of the SAPs will dictate their effectiveness for this purpose. In this paper, SAPs with different cross-linking degrees, particle sizes and amount of solubles are investigated. By varying these parameters, insight can be gained on the influence of each of these parameters on SAP properties such as the swelling capacity. In a next step, the SAPs can be implemented in mortar to assess their influence on mortar properties like workability, compressive strength or hydration kinetics. Based on these results, the ‘ideal’ SAP with tunable properties for a specific concrete application can be selected. For this purpose, an anionic SAP was synthesized with varying amounts of cross-linker and ground to particle sizes with d_50_ varying between 10 and 100 µm. The swelling capacity in demineralised water of 40 µm SAP particles increased with a decreasing degree of cross-linker from 66 g/g SAP with 1 mol% cross-linker to 270 g/g SAP in case of 0.15 mol% cross-linker, and was about three to four times larger than the swelling capacity in the prepared cement filtrate. The SAPs were tested for their effect on mortar workability, cement hydration kinetics and mechanical properties of the hardened mortar. With proper compensation for the absorbed water by the SAPs, the mortar workability was not negatively affected and the reduction in flow over the first two hours remained limited. The SAPs with the lowest swelling capacity, resulting in the smallest total amount of macro pores formed, showed the smallest negative effect on mortar compressive strength (a reduction of 23% compared to the reference after 28 days for an addition of 0.5 m% SAP) and a negligible effect on cement hydration. The difference in strength with the reference decreased as a function of mortar age. When using SAPs with particle sizes in the range of 10–100 µm, no significant differences between the studied particle sizes were found concerning the mortar properties. With the ease of upscaling in mind, the need to purify the SAPs and to remove the non-cross-linked soluble fraction was further investigated. It was shown that the solubles had no effect on the mortar properties, except for increasing the setting time with almost 100%.

## 1. Introduction

Superabsorbent polymers (SAPs) are 3D polymer networks that can retain large amounts of liquids, up to a thousand times their own dry weight, forming a hydrogel without dissolving [1]. This special feature makes SAPs very interesting to be used in concrete applications for different reasons [1]: mitigating autogenous shrinkage [2,3,4], modifying the rheology of fresh concrete [2,3], self-sealing and self-healing of cracked concrete [5,6,7,8,9,10,11], increasing the freeze-thaw resistance of concrete [12,13] etc. Hence, they can help to increase the durability of concrete structures in different ways. 

Various types of SAPs are currently available. On a chemical base, two extreme types can be distinguished: ionic (charged) and non-ionic (neutral) SAPs [14]. Commercially available SAPs are mostly ionic polymers and show higher absorption capacity. Another distinction can be made based on the type of cross-links occurring between the polymer chains: covalent, ionic or hydrogen bonding. For application in mortar or concrete, covalent cross-links are preferred as they are stable and strong [14]. These cross-links are the most essential part of the SAPs, as these bonds prevent the SAPs from dissolving. In the case of highly concentrated solutions of acrylic acid, self-crosslinking through hydrogen bonding can occur, although it has been found that these SAPs have a lower water absorption compared to polymers with the participation of the crosslinking agent and covalent bonds [15]. 

In addition to the aforementioned different properties of SAPs, the following parameters can also be used to distinguish SAPs: type (synthetic, semi-synthetic, natural), cross-linking degree, polymerization technique (bulk, suspension), particle size, etc. 

A decrease in swelling ratio is expected when the crosslinking density increases for SAPs submerged in deionized water [16,17] or in calcium rich solutions [18]. This result is logical as the cross-links will impede the SAPs from swelling. However, a certain amount of crosslinking is always needed as otherwise the material would dissolve upon exposure to liquids.

SAPs with varying particle sizes have already been implemented in mortar or concrete: 100 µm [19], 125–150 µm [20], 125–250 µm [3], 100–500 µm [21], 106–425 µm [16], 425–850 µm [16] and even up to some mm [22]. For mitigating autogenous shrinkage in cementitious materials, smaller SAP particles (d_50_ around 100–150 µm) are used [19,20], whereas for self-sealing and self-healing larger particles, sizes up to 500 µm are often reported in literature [5,7,22]. A smaller particle size is expected to have a smaller swelling capacity due to the smaller active circumference of the SAP compared to the bulk volume [21], although other researchers state the opposite: smaller SAPs will have a larger swelling capacity due to their larger surface area [22,23,24]. However, several researchers state that the particle size does not have an influence on the swelling capacity [16,25]. In this paper, SAPs with average dry particle size of 10, 40 and 100 µm were investigated. These particle sizes are smaller than the sizes studied in most published papers. However, it could be interesting to embed these SAPs in concrete, as their dimensions are in the same range as the other constituents in concrete like cement, fly ash or limestone. 

When synthesizing the SAPs, the possibility and ease of upscaling should be kept in mind, as (large) concrete structures will require a lot of SAPs. In this view, topics such as water consumption, need for purification, synthesis time, polymerization technique, etc. will play a role in deciding which SAPs will be suitable for application in concrete. Moreover, it is important to know whether the solubles or the fraction of the non-cross-linked polymer present in the final SAP have a negative effect on the mortar properties. This parameter is often expressed as its complement called the gel fraction. Most SAPs used in literature have a high gel fraction meaning that the amount of material that is chemically incorporated in the 3D network is high. In literature, the reported gel fractions vary from >80% [26], 95% [27,28] to nearly 100% [29]. The gel fraction of SAPs can be increased by removing unreacted particles via dialysis with demineralised water. However, this procedure is quite time and water consuming. Zohuriaan-Mehr states that one of the functional features of an ideal SAP material is a low soluble content and residual monomer [25]. In this study, it was investigated whether the presence (of a high number) of solubles (40% or more) has a negative effect on mortar properties. It could be interesting to know whether it is necessary to purify the SAPs and to remove the non-cross-linked soluble fraction or not, keeping the ease of upscaling in mind.

In this paper, SAPs with different cross-linking degrees, particle sizes and amount of solubles are investigated. By varying these parameters, insight can be gained on the influence of each of these parameters on SAP properties such as the swelling capacity. In a next step, the SAPs can be implemented in mortar to assess their influence on mortar properties like workability, compressive strength or hydration kinetics. Based on these results, the ‘ideal’ SAP with tuneable properties for a specific concrete application can be selected. 

## 2. Materials and Methods

### 2.1. Materials

A reference mortar mixture with a water-to-cement ratio of 0.43 was composed of ordinary Portland cement (CEM I 52.5 N, 510 kg/m³, HOLCIM), silica sand 0/2 (1530 kg/m³) and tap water (219.3 kg/m³). A constant dosage of 0.25 m% (of cement weight) polycarboxylate superplasticizer (Glenium 51, conc. 35%, BASF) was added to obtain an initial flow of 20 cm directly after mixing. In the case of SAP-containing mixtures, a constant amount of 0.5 m% SAPs by cement weight was added, as well as additional water to compensate for the water uptake by the SAPs. This additional water should be released after mortar setting as internal curing water but should not be included in the effective w/c-ratio (and is therefore shown separately in Table 1). The amount of additional water was 1.5 times the swelling capacity in cement filtrate obtained from a filtration test. This amount was based on previous tests performed by the author and was found to be the average amount needed to keep the consistency of the mortar mixtures constant. The amount of 0.5 m% SAPs by cement weight was chosen as it is an amount that lies in the range used and studied for internal curing (0.2–0.7 m%) [3,17,30,31] and for self-healing (0.5–2 m%) [5,32,33,34].

The studied SAPs were developed and produced by the Belgian chemical R&D company ChemStream bvba. The SAPs were formed through a copolymerization of sodium vinyl sulfonate (SVS) with 2-acryloylamino-2-methyl-propane-1-sulfonate (NaAMPS) using potassium persulfate (KPS) as thermal initiator and were obtained by a radical bulk polymerization reaction carried out at 70 °C. Five different amounts of the cross-linker *N,N*′-methylenebisacrylamide (MBA) (i.e., 0.15, 0.38, 0.575, 0.775 and 1.0 mol% with respect to the monomer) were used in order to obtain SAPs with different swelling capacities, as indicated in Table 1. The obtained SAPs were ground to particle sizes with d_50_ of 10, 40 and 100 µm using a RETSCH ZM200 centrifugal mill.

To prepare the SAP-containing mortar mixtures, first the dry components (cement and SAPs) were mixed for 30 s at low speed (140 rpm) to ensure a homogeneous distribution of the SAPs in the cement. The rest of the mixing procedure was performed according to the standard NBN EN 196-1 [35].

0–30 s: addition of water and superplasticizer while mixing at low speed (140 rpm);
30–60 s: addition of sand while mixing at low speed (140 rpm);
60–90 s: increasing mixing speed to 285 rpm;
90–120 s: scraping the edges of the bowl;
120–180 s: rest;
180–240 s: further mixing (speed 285 rpm).

In this paper, three different test series were made to study three different influences:

- Series 1: the influence of the degree of cross-linking;
- Series 2: the influence of the particle size;
- Series 3: the influence of the presence of solubles.

For the reference mixture, the code (see Table 1) is REF. For the mixes with SAPs, the code starts with CS (CS for ChemStream), followed by the amount of cross-linker used in mol% and ending with the particle size in µm (i.e., 10, 40 or 100). 

At the age of two days, all specimens (nine prisms of 40 × 40 × 160 mm³ for every mixture) were demoulded, wrapped in plastic foil and subsequently stored in an air-conditioned room with a relative humidity of 60 ± 5% and a temperature of 20 ± 2 °C until the age of testing.

With the ease of upscaling in mind, the need to purify the SAPs and remove the non-cross-linked soluble fraction was investigated. For this purpose, three more SAP types were synthesized by ChemStream with different amounts of solubles: 100%, 40% and 10% solubles. The amount of cross-linker was 0 mol% for the SAP with 100% solubles. For the other two SAPs with 40% and 10% solubles, the amount of cross-linker was 0.15 mol%. This time the code (see Table 2) starts with CS (from ChemStream) followed by SOL (from solubles) and ends with the amount of solubles present in the SAPs (i.e., 100%, 40% or 10%).

### 2.2. Characterisation of SAPs

The synthesized SAPs were characterised by the amount of solubles (i.e., the percentage of the non-cross-linked polymer fraction after synthesis), the swelling capacity in both demineralized water and cement filtrate solution and the mean particle size (d_50_) of the ground SAPs.

#### 2.2.1. Amount of Solubles

To determine the fraction of the non-cross-linked polymer or the amount of solubles, the following procedure was followed [25]. An excess of water was added to the SAP particles after they were dried in an oven at 80 °C. After 24 h, when the SAPs had reached their maximum swollen state, the swollen hydrogel was filtered from the excess of water. Next, the hydrogel was dried in an oven at 80 °C to constant mass. The difference in weight between the dried SAP after swelling (M_1_ [g]) and the initial dry SAP before starting the swelling procedure (M_0_ [g]) was measured. The % solubles was then calculated according to Formula (1):

(1) $$\%\;\mathrm{solubles}\;=\;\frac{\left(M_{0}-{\;M}_{1}\right)}{M_{0}}\;\times \;100$$

#### 2.2.2. Swelling Capacity

The swelling capacity of the SAPs in demineralized water and cement filtrate solution was determined by the filtration method described in the RILEM TC-RSC recommendation [36]. Therefore, approximately 100 g of fluid was added to around 0.15 g dry, unpurified SAP particles (exact mass of fluid and SAPs to be recorded). After 24 h, the SAP particles definitely reached their equilibrium swelling and everything was filtered using filter paper with a particle retention size of 12–15 µm. In order to exclude possible absorption of the fluid by the filter paper, the latter was first saturated with the testing fluid. To minimize evaporation during the test, the test setup was covered with a lid. After filtration, the amount of fluid that was not absorbed by the SAPs, was recorded. The amount of solubles that had passed the filter and was therefore present in the non-absorbed fluid was not subtracted as this amount is negligible compared to the total amount of non-absorbed fluid. The test was performed in triplicate. 

The cement filtrate was obtained by mixing 100 g Portland cement (CEM I 52.5 N) in one litre of demineralized water for at least 24 h, followed by filtration with filter paper with a particle retention size of 12–15 µm. The swelling ratio, i.e., the amount of fluid that can be absorbed by 1 g of unpurified SAPs can be calculated by formula (2):

(2) $$\mathrm{Swelling}\;\mathrm{ratio}\;[g\;\mathrm{fluid}/g\;\mathrm{unpurified}\;\mathrm{SAP}]\;=\;\frac{w_{\mathrm{fluid}\;\mathrm{added}}{\;-\;w}_{\mathrm{fluid}\;\mathrm{not}\;\mathrm{absorbed}}}{w_{\mathrm{dry}\;\mathrm{SAP}}}$$

with

w_fluid added_ [g]: the amount of fluid before filtration;
w_fluid not absorbed_ [g]: the amount of fluid that was not absorbed by the SAPs;
w_dry SAP_ [g]: the amount of dry, unpurified SAPs.

To take into account the rather high amount of solubles (>30%), or in other words a rather low gel fraction (<70%), of some of the synthesized SAPs, the swelling capacity was also recalculated taking into account the amount of non-cross-linked SAPs. These calculated values for the swelling capacity, expressed in g fluid per g SAP, express the amount of fluid that 1 g of purified SAPs (thus without any solubles) could absorb. In this way, it is possible to compare the swelling capacities of the SAPs studied in this paper with other SAPs mentioned in literature with low amounts of solubles.

#### 2.2.3. Mean Particle Size

The particle size distribution of the ground SAP powders was determined using a Malvern Mastersizer 2000 instrument that measures the size distribution of particles in the wet state by laser diffraction. Measurements were obtained by adding ethyl acetate to the dry SAP powders in a vial, stirring with caution in a sonicating bath and then transferring those with a pipet into the measuring system that was already filled with ethyl acetate as the solvent medium. Ethyl acetate was chosen as the SAPs under investigation do not swell in this fluid. The mean value (d_50_) of the particle size distribution is represented in µm.

### 2.3. Initial Flow and Flow over Time

The workability of the fresh mortar mixture was measured by a flow test according to the standard NBN EN 1015-3 [37]. The test was executed directly after mixing (i.e., approximately 4 min after cement came in contact with water), 30 min, 60 min, 90 min and 120 min after mixing. Before performance of the flow test, the mixture was first mixed for 30 s at low speed.

### 2.4. Hydration Kinetics

The hydration kinetics of the mixtures were measured with a TAM AIR isothermal heat conduction calorimeter (TA instruments). For this test, ampoules with 14 g of mortar were filled. As the components were mixed manually outside the calorimeter, the first hydration peak was not fully registered and will not be further analysed. The isothermal calorimetric tests were performed at 20 °C for 7 days. The data from the calorimetric tests were processed and converted to obtain the heat production rate q (J/g/h).

### 2.5. Flexural and Compressive Strength

The flexural strength was determined by means of a three-point-bending test on three mortar prisms with dimensions of 40 × 40 × 160 mm³. The compressive strength was measured on the halves resulting from the bending test. Both tests were performed according the standard NBN EN 196-1 [35] with the testing machine Walter + Bai DB 250/15. The mechanical properties were tested at 3, 7 and 28 days after casting. 

## 3. Results & Discussion

### 3.1. Influence of the Cross-Linking Degree

**Figure 1:**
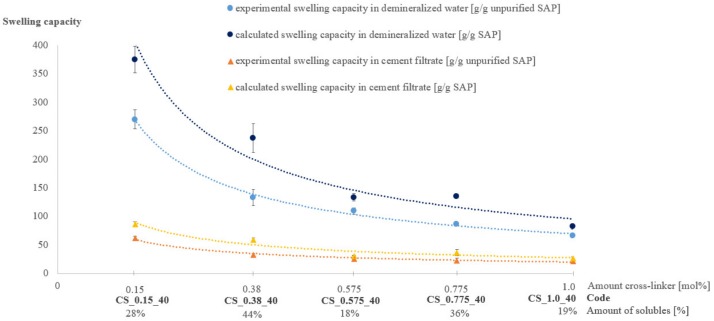
Characterization of superabsorbent polymers (SAPs): amount of solubles, experimental [g/g unpurified SAP] and calculated [g/g SAP] swelling capacity in demineralized water and cement filtrate solution (n = 3), Series 1.

SAPs with five different amounts of cross-linker (mol%) were produced, namely 0.15 mol%, 0.38 mol%, 0.575 mol%, 0.775 mol% and 1.0 mol%. The measured amounts of solubles for these SAPs were respectively 28%, 44%, 18%, 36% and 19% and were, compared to other literature [27,28,29,30], quite high. The higher the amount of cross-linker, the lower the experimental swelling capacity obtained through a filtration test, as indicated in Figure 1. The SAP with the highest amount of cross-linker (CS_1.0_40 with 1.0 mol% cross-linker) had a swelling capacity in demineralised water of 66 ± 4 g/g SAP while the SAP with the lowest degree of cross-linker (CS_0.15_40 with 0.15 mol% cross-linker) had a four times higher swelling capacity in demineralised water (270 ± 17 g/g SAP). This result was logical as the crosslinks will impede the SAPs from swelling. The same trend is seen for the swelling in cement filtrate solution and this is in accordance with findings reported earlier in literature [16,17,18]. The results of all SAPs show that their swelling capacities in cement filtrate solution are much lower compared to their swelling capacity in demineralized water. Cations like K^+^, Na^+^, Mg^2+^ or Ca^2+^ present in cement filtrate solution, give rise to a so-called charge screening effect of the negatively charged polymer chains, resulting in a lower repulsion of chains and a lowered fluid absorption and less swelling of the SAP particles. Furthermore, the presence of divalent cations like Ca^2+^ gives rise to an additional reduction of the swelling properties as these cations form strong complexes with the sulfonate groups and can therefore act as cross-linkers [16,33,38,39]. 

When looking at the calculated values of the swelling capacity that take into account the amount of solubles (Figure 1), it can be seen that two mixtures do not follow the expected trend, namely CS_0.38_40 and CS_0.775_40. Both SAPs show a higher calculated swelling capacity than expected, due to their high amount of solubles of 44% and 36% for CS_0.38_40 and CS_0.775_40, respectively.

**Figure 2:**
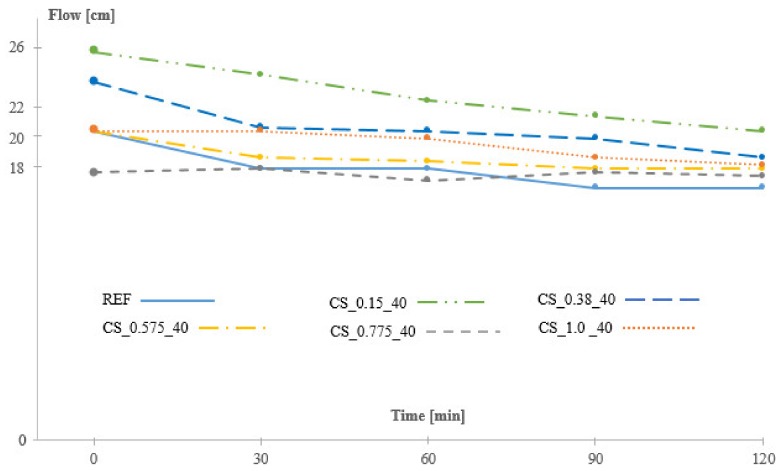
Initial flow and flow over time of the reference mixture and the mixtures containing SAPs with different amounts of cross-linker, Series 1.

In a case where no additional water is added to the mixture, the workability would be negatively affected as the SAPs would absorb part of the mixing water. Therefore, it was decided to add an amount of additional water of 1.5 times the experimentally obtained swelling capacity in cement filtrate from the filtration test, based on previous tests using similar SAPs. The initial flow of the reference mixture was 205 mm and decreases after 120 min to 168 mm. The initial flow and flow over time of the reference and the five SAP-containing mixtures can be seen in Figure 2.

With the aforementioned amount of additional water, two mixtures containing SAPs, namely CS_0.575_40 and CS_1.0_40 show similar workability in time as the reference. Their reduction in flow over the first two hours was limited and somewhat smaller than what was noticed for the reference mixture without SAPs. The SAP containing mixtures CS_0.15_40 and CS_0.38_40 however, showed a larger initial flow, meaning that the amount of additional water was not fully absorbed by the SAPs immediately. These two SAPs contained the smallest amount of cross-linker, respectively 0.15 mol% and 0.38 mol% and showed the largest deviation in swelling capacity, as can be seen in Figure 1. For mixture CS_0.775_40 the initial flow is somewhat lower than for the reference mixture (17.5 cm instead of 20.5 cm). For this mixture, the amount of additional water was underestimated, and the SAPs absorbed part of the mixing water, resulting in a lower initial flow. However, when looking at the flow values after 120 min, it can be seen that all the mixtures have a similar flow in the range 18–22 cm. The reference mixture without SAPs has even the lowest flow of 16.75 cm after 120 min. Differences in swelling kinetics between the different SAPs could be a reason for the delayed water uptake by the SAPs and the varying workability over time.

From these results, it can be seen that the correct amount of additional water is not that straightforward to determine. The correct amount of additional water, in order to obtain the same workability as the reference mixture, should be determined for each SAP separately by a trial-and-error procedure, starting from the swelling capacity in cement filtrate solution obtained from a filtration test. Not only the initial flow should be taken into account, but also the flow after some time, for example after 120 min, should be taken into account as different SAPs will have different swelling kinetics. Although this is a time-consuming method, it is very important to add the correct amount of additional water as an over-or underestimation of this amount will change the W/C ratio of the mixture, which will have a significant influence on the test results.

**Figure 3:**
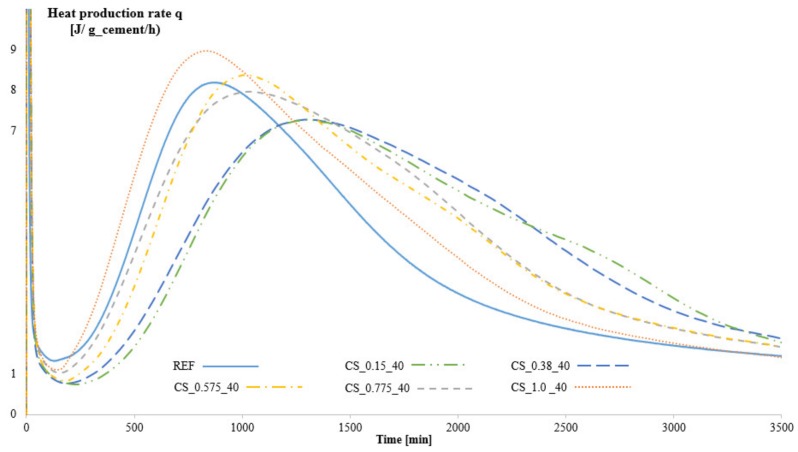
Hydration kinetics of the reference mixture and the mixtures containing SAPs with different amounts of cross-linker, Series 1.

Figure 3 shows the heat production rate as a function of time. It can be seen that the hydration of the mixture containing SAPs and with the correct amount of additional water and thus approximately the same W/C ratio as the reference (namely CS_0.575_40) is slightly retarded with 2.5 h compared to the reference mixture as indicated by the small shift to the right and by the reduction in maximum heat flow values. The SAPs with the lowest swelling capacity, i.e., CS_1.0_40 with 1.0 mol% cross-linker, show a negligible effect on cement hydration. A retardation in setting time caused by the addition of SAPs was already reported by several authors [40,41,42,43]. However, the mixtures with SAP that show a slower absorption of the additional water, i.e., CS_0.15_40 and CS_0.38_40, show a much larger delay in the setting time of 6.5 h compared to the reference, as the actual W/C ratio of these mixtures is higher (and unwanted) compared to the reference [44]. Therefore, the delay in setting time for these mixtures is caused by a combination of the higher W/C ratio and the presence of the SAPs. A premature release of stored water may also have caused this.

**Figure 4:**
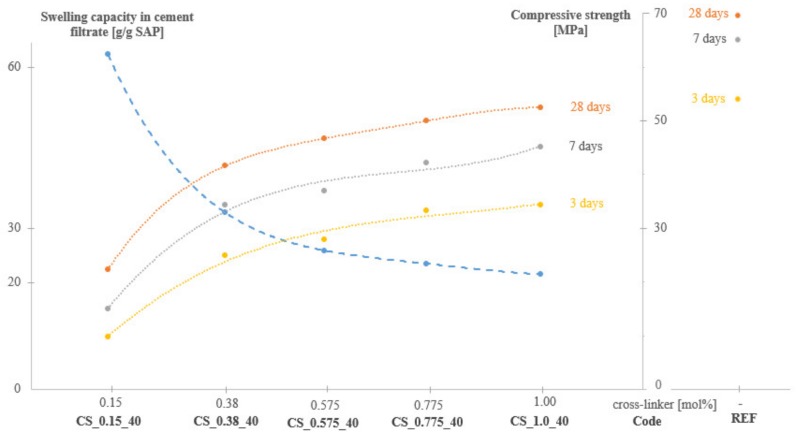
Swelling capacity in cement filtrate and mortar compressive strength (n = 6) after 3, 7 and 28 days for SAPs with different amounts of cross-linker, compared to the reference.

The compressive strength of the different mixtures after 3, 7 and 28 days is depicted in Figure 4, together with the experimentally obtained swelling capacity in cement filtrate of the unpurified SAPs. Two main conclusions can be made based on these results:

(1) The higher the swelling capacity (i.e., the lower the amount of cross-linker), the lower the compressive strength. This result is logical as the swollen SAPs will create macro pores in the matrix, negatively affecting the compressive strength compared to the reference [1,2,3,45]. After 28 days, the compressive strength of SAP CS_0.15_40 (i.e., with the lowest amount of cross-linker) was 67% lower than the value noticed for the reference. SAP CS_1.0_40 (i.e., with the highest amount of cross-linker) had a compressive strength at that age which was 23% lower than what was noticed for the reference.
(2) The difference in strength with the reference decreases as a function of the mortar age. For example, for SAP CS_1.0_40 this difference decreased from −35% after 3 days to −29% after 7 days and to −23% after 28 days compared to the reference. This trend was observed for all the SAPs with different cross-linking degrees.

For the flexural strength at 3, 7 and 28 days similar trends were found. After 28 days, the flexural strength of SAP CS_0.15_40 (i.e., with the lowest amount of cross-linker) was 4.5 ± 0.2 MPa, which was 52% lower than the value noticed for the reference of 9.5 ± 0.5 MPa. SAP CS_1.0_40 (i.e., with the highest amount of cross-linker), and had a flexural strength at that age which was 26% lower than what was noticed for the reference. Also, the difference in flexural strength with the reference decreased as a function of the mortar age. 

### 3.2. Influence of Particle Size

**Figure 5:**
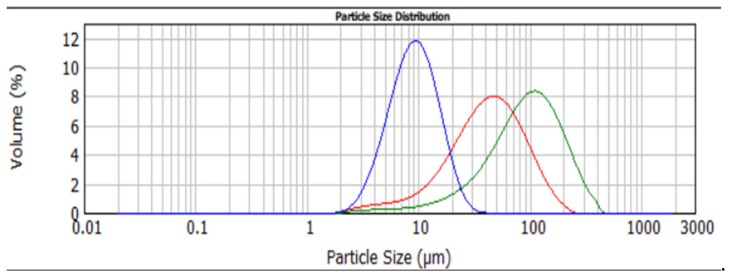
Particle size distribution of CS_1.0: CS_1.0_10 (blue), CS_1.0_40 (red) and CS_1.0_100 (green).

In a second test series, the influence of different particle sizes was investigated. For this purpose, the SAPs from Series 1 resulting in the lowest strength reduction and with the smallest effect on the hydration rate were selected, namely CS_0.775 (0.775 mol% cross-linker) and CS_1.0 (1.0 mol% cross-linker). These SAPs were ground to particle sizes with d_50_ of 10, 40 and 100 µm. The particle size distributions of the SAP CS_1.0 with mean particle size 10, 40 and 100 µm are depicted in Figure 5.

Similar tests as for test Series 1 were performed. The results are summarized in Table 3 and Table 4. The results of the SAP CS_1.0 with different particle sizes of 10, 40 and 100 µm were not significantly different (α = 0.05) concerning the swelling capacity in both demineralised water and cement filtrate solution (Table 3) and mortar compressive strength after 28 days (Table 4). The swelling capacity in demineralised water for CS_0.775_40 was found to be significantly different from the swelling capacity of SAPs with particle sizes of 10 µm and 100 µm (Table 3). It must be noted that the swelling capacity of the SAPs with particle sizes of 40 µm was not measured at the same time as for the particle sizes of 10 and 100 µm. As a result, differences in temperature, solution or other conditions could have had an influence on the results. In cement filtrate solution, the swelling capacity was not significantly different (α = 0.05) between the studied particle sizes for SAP CS_0.775 (Table 3). This is in accordance with findings in literature [16,25]. Also, the mortar compressive strength at 28 days was not significantly different between the studied particle sizes for this SAP (Table 4). 

From these results it can be concluded that a change in particle size in the range of 10–100 µm does not have a significant influence on the studied parameters.

In Table 4, the importance of determining the correct amount of additional water based on both the initial flow and the flow in time can be seen. The initial flow of mixtures CS_0.775_40 and CS_1.0_40 was remarkably lower than the flow of the other SAP-containing mixtures. However, the same remark as earlier must be made that the tests with SAPs with particle size of 40 µm were not conducted at the same time as the tests on SAPs with particle sizes of 10 and 100 µm. Although several mixes showed a much larger initial flow compared to the reference, this difference disappears after 120 min and all the mixtures had a similar flow in the range of 18–19 cm. 

### 3.3. Influence of Solubles

With the ease of upscaling in mind, the need to purify the SAPs and remove the non-cross-linked soluble fraction was further investigated. For this purpose, three more SAP types were synthesized by ChemStream with different amounts of solubles: 100%, 40% and 10% solubles. The d_50_ of these SAPs were respectively 20 µm, 100 µm, 30 µm. From the previous paragraph, it was proven that the particle size did not have a significant influence on the results.

From Table 5 and Table 6 it can be seen that the lower the amount of solubles, the higher the swelling capacity and as a consequence the lower the compressive strength. Although the SAP CS_SOL_100% was made without any cross-linker, this SAP showed a very limited swelling capacity in demineralized water and cement filtrate solution. The Ca^2+^ ions present in cement filtrate solution can form strong complexes with the sulfonate groups of the SAPs and can therefore act as cross-linkers, resulting in a limited swelling capacity, even in the absence of cross-linker.

**Figure 6:**
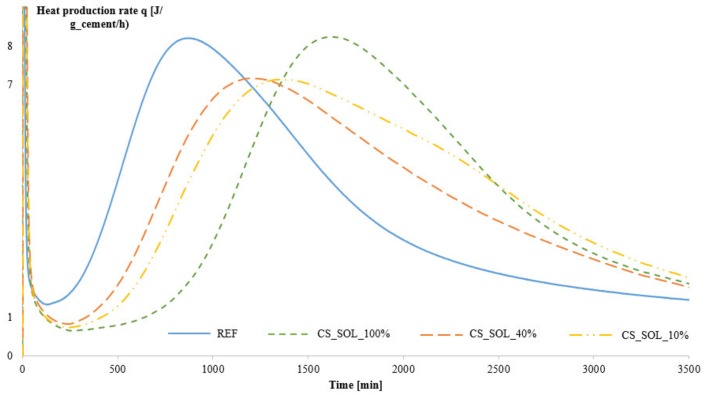
Hydration kinetics of the reference mixture and the mixtures containing SAPs, different amount of solubles (Series 3).

However, the presence of solubles itself has no effect on the studied mortar properties as CS_SOL_100% shows similar results compared to the reference mixture, see Table 6. The only effect of the amount of solubles can be seen when investigating the hydration kinetics, as the graph for CS_SOL_100% has shifted to the right, meaning an increase of the setting time, see Figure 6. The peak in the heat production rate shifts from 840 min (i.e., 14 h) for the reference mixture to almost the double amount of time of 1635 min (around 27 h) for the mixture CS_SOL_100%. 

It can be concluded that extra purification of the SAPs is not needed as the presence of solubles in unpurified SAPs does not negatively affect the studied mortar properties. It was therefore confirmed that omitting the extra purification step for the SAPs used in Series 1 and Series 2 had no negative effect. 

However, from an economical point of view, it could be interesting to purify the SAPs in order to have the most efficient SAP addition in the concrete. In the case of unpurified SAPs, part of the added SAP will not be active and will thus not have any benefit in the concrete. In case of purification of the SAPs, the water and energy consumption and drying time must be optimized in order to still be economical interesting.

The possibility, ease and cost-effectiveness of purifying the SAPs should be considered when upscaling the SAP production.

## 4. Conclusions

The following conclusions can be drawn based on the results of this study:

- The swelling capacity of SAPs can be fine-tuned by varying the cross-linking degree of the SAPs.
- The correct amount of additional water to obtain the same workability as the reference mixture should be determined for each SAP separately, starting from the swelling capacity in cement filtrate solution obtained from a filtration test. Not only the initial flow directly after mixing should be taken into account, but also the flow after 120 min as differences in swelling kinetics of the SAP could delay the water uptake by the SAPs.
- The hydration of mixtures containing SAPs is retarded with 2.5 to 6.5 h compared to the reference mixture.
- The higher the swelling capacity of the SAP (i.e., the lower the cross-linking degree), the lower the compressive strength of the mortar. This result is logical as the swollen SAPs will create macro-pores in the matrix, negatively affecting the compressive strength compared to the reference.
- The difference in compressive strength with the reference decreases as a function of the mortar age.
- The SAP particle size (d_50_ = 10, 40 and 100 µm) has no significant effect on the studied mortar properties (swelling capacity in cement filtrate, flow after 120 min and compressive strength after 28 days).
- The presence of solubles has no negative effect on the mortar properties as CS_SOL_100% shows similar results concerning the flow and compressive strength compared to the reference mixture. The only effect of the presence of solubles on mortar properties is an increase of the setting time. It can be concluded that extra purification of the SAPs is thus not needed from this point of view. A correction should be made on the amount to be added to the mixture.

## 5. Future Work

In the present article, the main parameters influencing autogenous shrinkage were studied. The authors believe that a preparatory study covering these parameters will result in a better understanding of and insight in the principles of mitigating autogenous shrinkage with SAPs. In a next step, the used SAPs will be implemented in cementitious materials to study their effect on mitigating autogenous shrinkage.

## Figures and Tables

**Table 1:** Studied mortar mixtures with their code, extra water added and corresponding water-to-cement ratios (additional and total) for Series 1 and 2.

Code	Extra Water Added [kg/m³]	[w/c]_add_ (-)	[w/c]_ot_ (-)
REF	0	0	0.43
CS_0.15_40	238	0.47	0.90
CS_0.38_40	126	0.25	0.68
CS_0.575_40	99	0.19	0.62
CS_0.775_10	106	0.21	0.64
CS_0.775_40	90	0.18	0.61
CS_0.775_100	97	0.19	0.62
CS_1.0_10	87	0.17	0.60
CS_1.0_40	82	0.16	0.59
CS_1.0_100	85	0.17	0.60

**Table 2:** Studied mortar mixtures with their code, amount of cross-linker, additional water and corresponding water-to-cement rations (additional and total), series 3.

Code	Additional Water [kg/m³]	[w/c]_add_ (-)	[w/c]_tot_ (-)
CS_SOL_100%	9.82	0.02	0.45
CS_SOL_40%	75.71	0.15	0.58
CS_SOL_10%	157.10	0.31	0.74

**Table 3:** Characterization of SAPs: solubles, swelling capacity in demineralised water and cement filtrate (n = 3), series 2.

Code	Solubles [%]	Swelling Capacity in	
		Demineralised Water [g/g SAP]	Cement Filtrate [g/g SAP]
CS_0.775_10	30	75 ± 6	28 ± 1
CS_0.775_40	36	87 ± 2	23 ± 3
CS_0.775_100	30	79 ± 3	25 ± 2
CS_1.0_10	23	59 ± 1	23 ± 2
CS_1.0_40	19	66 ± 4	21 ± 3
CS_1.0_100	24	61 ± 2	22 ± 1

**Table 4:** Characterization of mortar: initial flow, flow after 120 min, mortar compressive strength after 3, 7 and 28 days (n = 6), Series 2.

Code	Initial Flow [cm]	Flow After 120 Min [cm]	Compressive Strength [MPa]		
			3 days	7 days	28 days
REF	20.5	16.75	53.0 ± 2.4	63.9 ± 0.7	68.4 ± 6.7
CS_0.775_10	28.5	19.75	23.8 ± 0.3	36.2 ± 1.1	48.4 ± 0.6
CS_0.775_40	17.75	17.5	33.3 ± 0.8	42.2 ± 2.6	50.0 ± 0.9
CS_0.775_100	26.25	18.75	24.2 ± 1.7	36.7 ± 0.6	49.1 ± 1.2
CS_1.0_10	26.75	19.75	28.7 ± 0.6	42.2 ± 0.4	53.2 ± 1.1
CS_1.0_40	20.5	18.25	34.3 ± 1.3	45.1 ± 1.9	52.5 ± 0.8
CS_1.0_100	26	19.75	32.2 ± 2.1	44.8 ± 2.0	55.5 ± 2.1

**Table 5:** Characterization of SAPs: solubles (%), swelling capacity in demineralised water and cement filtrate (g/g SAP), n = 3, Series 3.

Code	Solubles [%]	Swelling Capacity in	
		Demineralised Water [g/g SAP]	Cement Filtrate [g/g SAP]
CS_SOL_10%	10	382.17 ± 14.58	92.41 ± 7.45
CS_SOL_40%	40	224.93 ± 9.20	44.54 ± 2.62
CS_SOL_100%	100	1.43 ± 0.32	5.78 ± 1.73

**Table 6:** Characterization of mortar: flow after 120 min [cm], compressive strength after 3, 7 and 28 days [MPa], n = 6, Series 3.

Code	Flow After 120 Min [cm]	Compressive Strength [MPa]		
		3 days	7 days	28 days
REF	16.75	53.03 ± 2.35	63.94 ± 0.71	68.35 ± 6.7
CS_SOL_10%	10.5	15.19 ± 0.31	21.71 ± 1.33	28.62 ± 1.72
CS_SOL_40%	13	24.99 ± 1.1	34.64 ± 0.77	43.32 ± 0.40
CS_SOL_100%	16	49.90 ± 8.27	64.24 ± 1.45	77.1 ± 3.82
